# Insights from incorporating quantum computing into drug design workflows

**DOI:** 10.1093/bioinformatics/btac789

**Published:** 2022-12-07

**Authors:** Bayo Lau, Prashant S Emani, Jackson Chapman, Lijing Yao, Tarsus Lam, Paul Merrill, Jonathan Warrell, Mark B Gerstein, Hugo Y K Lam

**Affiliations:** HypaHealth, HypaHub Inc., San Jose, CA 95128, USA; Program in Computational Biology and Bioinformatics, Yale University, New Haven, CT 06520, USA; Department of Molecular Biophysics and Biochemistry, Yale University, New Haven, CT 06520, USA; Program in Computational Biology and Bioinformatics, Yale University, New Haven, CT 06520, USA; Department of Molecular Biophysics and Biochemistry, Yale University, New Haven, CT 06520, USA; HypaHealth, HypaHub Inc., San Jose, CA 95128, USA; HypaHealth, HypaHub Inc., San Jose, CA 95128, USA; HypaHealth, HypaHub Inc., San Jose, CA 95128, USA; Program in Computational Biology and Bioinformatics, Yale University, New Haven, CT 06520, USA; Department of Molecular Biophysics and Biochemistry, Yale University, New Haven, CT 06520, USA; Program in Computational Biology and Bioinformatics, Yale University, New Haven, CT 06520, USA; Department of Molecular Biophysics and Biochemistry, Yale University, New Haven, CT 06520, USA; Department of Computer Science, Yale University, New Haven, CT 06520, USA; Department of Statistics & Data Science, Yale University, New Haven, CT 06520, USA; HypaHealth, HypaHub Inc., San Jose, CA 95128, USA

## Abstract

**Motivation:**

While many quantum computing (QC) methods promise theoretical advantages over classical counterparts, quantum hardware remains limited. Exploiting near-term QC in computer-aided drug design (CADD) thus requires judicious partitioning between classical and quantum calculations.

**Results:**

We present HypaCADD, a hybrid classical-quantum workflow for finding ligands binding to proteins, while accounting for genetic mutations. We explicitly identify modules of our drug-design workflow currently amenable to replacement by QC: non-intuitively, we identify the mutation-impact predictor as the best candidate. HypaCADD thus combines classical docking and molecular dynamics with quantum machine learning (QML) to infer the impact of mutations. We present a case study with the coronavirus (SARS-CoV-2) protease and associated mutants. We map a classical machine-learning module onto QC, using a neural network constructed from qubit-rotation gates. We have implemented this in simulation and on two commercial quantum computers. We find that the QML models can perform on par with, if not better than, classical baselines. In summary, HypaCADD offers a successful strategy for leveraging QC for CADD.

**Availability and implementation:**

Jupyter Notebooks with Python code are freely available for academic use on GitHub: https://www.github.com/hypahub/hypacadd_notebook.

**Supplementary information:**

Supplementary data are available at *Bioinformatics* online.

## 1 Introduction

Rapid advancement of biotechnologies is enabling an unprecedented rate of data generation in the biomedical domain, with game-changing applications if we can harness even some of the multi-scale, many-body biological complexity. Computational biology has provided critical contributions to our understanding of this complexity, in problems, such as protein folding, genome assembly, variant detection and many others. Core computational biology algorithms have thus far been successful in pushing the limit of classical computers, with horizontal scaling across multiple computing units limited mainly by network speed, and vertical scaling in a single computing unit limited by Moore’s Law. One important application of biotechnology is drug discovery, with costly experimental work benefiting from, e.g. the lead search capabilities of computer-aided drug design (CADD). For such combinatorial, many-body problems, modern techniques are limited by computational hardware in both the information content of a model as well as the system size for a given model that can be computed with reasonable computational resources (Sliwoski *et al.*, 2014).

Quantum computing (QC) provides an alternative computing paradigm. Quantum algorithms promise efficient solutions to problems that are difficult through classical computing (Feynman, 1982 (quantumalgorithmzoo.org), and could be applicable to complicated biochemical systems. Although general-purpose, fault-tolerant, large-scale QC technologies are still in the works, QC technology has already enabled promising simulations of non-trivial Hamiltonians (Smith *et al.*, 2019). Current noisy intermediate-scale quantum-era (Preskill, 2018) hardware, while significantly limited by noise and short coherence time, is available even through cloud service providers (aws.amazon.com/braket; azure.microsoft.com/en-us/services/quantum). While practical quantum supremacy is still under debate, there is certainly enough evidence that QC could have advantages over analogous classical computing in some aspects in the near future. In fact, many institutions have started to explore QC as applied to drug discovery, with near-term focuses on selected tasks, such as lead optimization or compound screening (Zinner *et al.*, 2021).

Many of the advantages of QC are related to the fact that quantum bits, or qubits, provide a state space that is exponentially larger than that of classical counterparts. Thus, while a classical bit has two discrete states, 0 and 1, a qubit can be treated as a rotatable vector that can be represented by two continuously varying angles. Moreover, by interfering with each other like waves, these qubits can take on joint configurations inaccessible to classical systems. These behaviors may lend QC advantages over classical computing in terms of speed, and potentially even representation of data (Emani *et al.*, 2021). However, currently available commercial quantum computers, based on the number of available qubits, limit the size of problems that can be mapped and solved. This is especially true for the logic-gate systems, which perform logical operations via sequential qubit-rotation gates. Available numbers of qubits are on the order of ∼100 (Ball, 2021) (azure.microsoft.com/en-us/services/quantum/; ibm.com/quantum-computing), at the higher end. Furthermore, many published QC algorithms were designed based on a putative quantum equivalent of RAM, or qRAM (Giovannetti *et al.*, 2008), where superpositions of qubits can be directly queried. The current lack of commercially available realizations of qRAM implies near-term use of QC algorithms has to rely on classical memory. As such, any real-world applications of QC would have to judiciously divide tasks between classical and QC capabilities. These ‘hybrid’ approaches would have to first be evaluated for feasibility (i.e. solving the problem to reasonable accuracy), and subsequently for potential quantum advantages over purely classical equivalents. The question of feasibility is especially important in biological problems where the structure of the data is complex, and has recently been considered for the case of mRNA codon optimization (Fox *et al.*, 2021).

Here, we explore the incorporation of QC into an otherwise classical computational screening workflow. We take this hybrid approach with two primary aims: (i) demonstrating how QC can be combined with classical computing to create a tool that solves real-world, multi-dimensional biomedical problems; and (ii) comparing multiple quantum machine learning (QML) algorithms to published classical counterparts, both in simulation and on actual quantum computers.

**Fig. 1.**
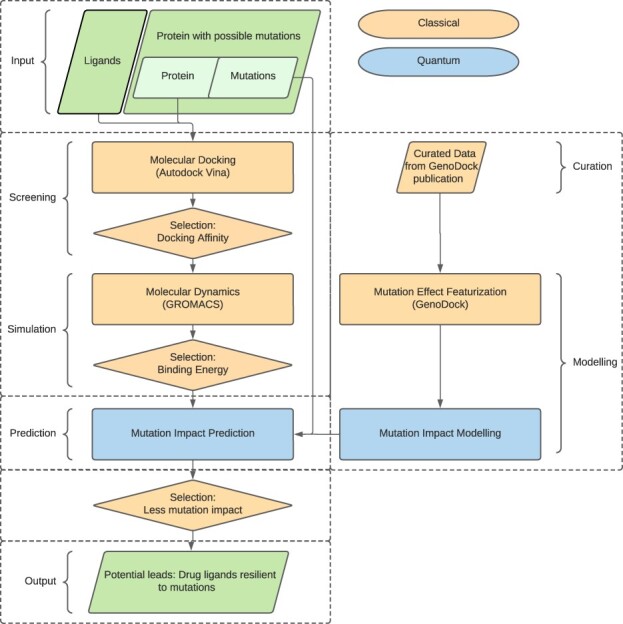
Given a set of ligands and a target protein with known or hypothetical mutations, the classical/quantum hybrid screening pipeline seeks for potential leads—ligands that would bind favorably to the protein as well as its mutants (green parallelograms, representing the input data and output results). The current implementation performs classical computation with down-selection after screening and simulation. Using a set of curated data, featurization is performed before modeling by QML (blue rectangles). The impact of mutations on the down-selected ligands is then by the QML (blue rectangles). Further down-selection based on mutation impact is performed classically. We note that the selection is effectively an optimization or ranking problem, which, along with docking and molecular simulation, could be considered to have a quantum computational approach in the future

Specifically, we demonstrate a hybrid computing approach, named HypaCADD, applied toward CADD. HypaCADD is a hyperscale computational pipeline that integrates large-scale genomics and protein structure data for drug discovery. It consists of highly computationally intensive applications, such as molecular docking, binding affinity prediction, molecular dynamics and machine-learning (ML)-based lead search and optimization. To prove the readiness and potential utility of QC in drug discovery research, the pipeline uses QML for predicting mutational effects on drug binding. It compares the quantum and classical computing results, and demonstrates the consistency between running in simulation and on real quantum computers from Rigetti (rigetti.com) and IBM (ibm.com/quantum-computing). We choose a straightforward form of hybridity: the data are preprocessed on a classical system and fed to quantum simulators and/or hardware. We identify an independent module of the workflow (Fig. 1) to convert to a quantum analogue, namely, the module associated with calculating the impact of amino acid mutations on ligand binding affinity. The advantages of this approach are 2-fold. First, we utilize the published results of a classical ML approach (Wang *et al.*, 2019) for careful validation of the quantum methods. Second, we completely replace this module in a tractable manner for current quantum simulators and hardware, thereby applying state-of-the-art QC to an important component of the drug-design pipeline. Downstream, we aim to start replacing, partially or even fully, some of the more computationally complex components, such as the molecular docking and molecular dynamics algorithms. As QC continues to advance, it can also be used for virtual screening with larger-size molecules or for more accurate and faster molecular dynamics simulation. For now, we find that many of the quantum algorithms we use for the mutation-impact module perform on par with their classical counterparts. HypaCADD thus affirms the value of leveraging a hybrid approach to make QC readily accessible and meaningful to biomedical scientists in solving challenging drug discovery problems.

This is a measured approach to the introduction of QC into the field of CADD. We prioritized the need for generating a pipeline immediately applicable to large-scale, real-world data for drug repurposing over the ultimate goal of fully exploiting putative advantages of QC. The possible advantages of QML, in particular, could be of the following forms (Schuld and Petruccione, 2018): advantages in computational complexity, which is a measure of the number of operations required to obtain a reasonably accurate solution (related to QC ‘speedup’); improvements in sample complexity, which is a measure of the number of data samples needed to generalize well from a training dataset; and improvements in model complexity, which is a measure of the expressivity of a model to represent the structure inherent in the data. There are several instances of particular algorithms with theoretical guarantees of scaling improvements over classical counterparts. However, in a general QML framework, especially hybrid classical-quantum models, the exact advantages of any approach depend on the component algorithms, datasets, associated noise and the number of sampling measurements required for robustness (Schuld and Petruccione, 2018). Our target is to use this work as a foundation to make the QML models in the CADD framework increasingly sophisticated, such that they might eventually provide heuristic evidence for QC advantages, without making strong claims about the relative advantages of the methods tested in the current work. Another consideration is that other modules in the docking/MD pipeline may be more inherently ‘quantum mechanical’ in nature, as well as more computationally intensive (and therefore more in need of QC speedup). One of the main focuses of current efforts in this direction is to improve quantum chemistry simulations, which would, in turn, improve the parameterization of force-fields, and also the *ab initio* quantum mechanical calculations of binding affinity (Cao, 2018). However, some of those advances will have to wait for both hardware growth and methodological developments to allow for high-throughput screening: the number of qubits required would depend on the size of the ligand and target binding pocket, and the hybrid classical-quantum frameworks would need to be carefully designed for near-term, noisy devices. Instead, several recent approaches to protein design and docking address the scale of the problem by contending with classical parameterizations of the problems: Mulligan *et al.* (2020) and Khatami *et al.* (2022) tackle protein sequence design using classical pairwise interaction potentials between amino acids as cost functions optimized on quantum annealers and logic-gate-based computers, respectively; Banchi *et al.* (2020) find ligand–protein docking solutions on a Gaussian Boson Sampler by representing the interface using a joint distance graph; and Li *et al.* (2021) use a reduced representation of ligands and receptors in hybrid classical-QML approaches, finding potential QC advantages. Our employment of QML is complementary to these strategies in employing classical parameters in a quantum model, with a view to targeting the important problem of mutational impact of ligand binding. We particularly foresee the promise of QML, as ML approaches, being agnostic to the underlying causal mechanisms, are often easily generalizable to other problems [see discussion in Cao (2018)]. For example, the numerical force-field fitting procedures in the design of programs, such as AutoDock Vina (Morris *et al.*, 2009; Trott *et al.*, 2010), involve optimization of parameters to minimize the deviation of predictions from known experimental structures. This can potentially be cast as a QML problem. We hope this generality can also be exploited by the CADD community. We further emphasize, however, that our goal here is not to definitively prove the quantum advantage of these models. The exact nature and degree of putative advantages in representation and/or speedup in general QML models are actively being debated by the scientific community. Our analysis is intended to lay out a clear path to QC incorporation, in the near-term and for high-throughput applications.

HypaCADD is a general-purpose workflow, applicable to any set of input drug ligands, target proteins and point mutations of the proteins. Here, we apply HypaCADD to the highly relevant problem of finding drug ligands that bind with high affinity to SARS-CoV-2 virus proteins, and whose binding is resilient against a spectrum of mutations in the target protein. Because of its importance to protein processing and viral replication, the main SARS-CoV-2 protease 3CL^*pro*^ has been frequently targeted (Jang *et al.*, 2021) for drug–ligand analyses. We use this protein and a set of associated amino acid mutations for our analyses. We initially applied a sequential virtual screening protocol, including molecular docking and molecular dynamics simulations, to 30 000 ligands and identified two potential drug candidates for the SARS-CoV-2 3CL^*pro*^ protein. Subsequently, we predict the effect of protein mutations that can guide lead search and optimization. Our high-throughput screening analysis will complement existing models that incorporate dynamic and structural information to assess the impact of variants on proteins and protein–ligand complexes (Torrens-Fontanals *et al.*, 2022). We believe our approach increases the robustness of anti-SARS-CoV-2 drug discovery.

## 2 Materials and methods

### 2.1 Datasets

The drug dataset (features: 3D, Ref/Mid pHs, Drug-like, In-stock) from ZINC in PDBQT format was downloaded from zinc.docking.org, comprising a total of more than 10 million compounds. The X-ray crystallized 3D structure in PDB format of 3CL^*pro*^ of HCoV-229E (PDB code: 2ZU2) (Lee *et al.*, 2009) and of 3CL^*pro*^ of SARS-CoV-2 of COVID-19 (PDB code 6W63) (Mesecar, 2020) were downloaded from rcsb.org. 2ZU2 was used as the receptor to validate our screening methodology in HCoV-229E and 6W63 was used as the receptor to identify lead compounds for SARS-CoV-2. Mutations in SARS-COV-2 on nsp5 3CL^*pro*^ were collected from covidcg.org.

### 2.2 Molecular docking

AutoDock Vina (Trott *et al.*, 2010) was chosen as the docking tool to conduct the initial virtual screening by estimating the non-covalent binding of receptors and ligands. The receptors were processed using a custom script, removing ‘HETATM’ components including ligands, ions and waters and protonating His41 to the neutral state at the epsilon nitrogen (N*ε*2). Then, the preparation of a receptor for docking was finished by a script in AutoDock Tools (Morris *et al.*, 2009) (‘prepare_receptor4.py’), where the polar hydrogens and Gasteiger charges were added and where the PDB files were converted to PDBQT format. The grid boxes for docking were centered on the active His41 residue and were set to extend 26 grid points in each direction. The lower the predicted binding affinity score is, the higher the binding affinity between the receptors and ligands.

### 2.3 Molecular dynamics simulation

Molecular dynamics were performed using a combination of GROMACS (Abraham *et al.*, 2015; Berendsen *et al.*, 1995; Hess *et al.*, 2008; Lindahl *et al.*, 2001; Páll *et al.*, 2015; Pronk *et al.*, 2013; Van Der Spoel *et al.*, 2005), AMBER03 force-field (Alvarado *et al.*, 2020; Bremer *et al.*, 2020; Duan *et al.*, 2003), Open Babel (O’Boyle *et al.*, 2011) and ACPYPE (Batista *et al.*, 2006; Sousa da Silva *et al.*, 2012; Wang *et al.*, 2004, 2006) with General Amber Force-Field and TIP3P model (see Supplementary Material). Shorter 0.4 ns simulations were performed for various ligands with different binding affinities estimated by AutoDock Vina (Trott *et al.*, 2010). Longer simulations (10 ns) were performed on selected ligands, including the ligand named X77 that was co-crystallized with SARS-CoV-2 3CL^*pro*^. After the simulation, GROMACS’s output is analyzed with gmx_mmPBSA (Miller *et al.*, 2012; Valdés-Tresanco *et al.*, 2021), which uses Ambertools 2.0’s MMPBSA to calculate binding free energy using the generalized Born surface area (GBSA) method.

### 2.4 Evaluation of viral mutations’ impacts on protein–drug interactions

A classical physical-statistical classifier (Wang *et al.*, 2019) was developed to predict the impacts of single nucleotide variants on protein–drug interactions. The classifier, GenoDock, uses genomic, structural and physicochemical features. As discussed in the Supplementary Material, this work reformulates the framework for practical treatment of non-human proteins with less annotations. The GenoDock features per mutation-ligand configuration include (feature names are italicized and in parentheses): amino acid side-chain volume change index (*volume_change_index*), polarity change index (*polarity_change_index*), distance between the mutation and drug ligand (*distance*), molecular weight (*molecular_weight*), H-bond donor (*H_bond_donor*) and acceptor counts (*H_bond_acceptor*), rotatable bond counts (*rotatable_bond*), polar surface area (*Polar_Surface_Area*) and whether the variant occurs in the ligand binding site or not (*bind_site*). For the case of human variants, we also included features that are related to the conservation of a variant and its frequency in human populations: allele frequency (*allele_freq*); SIFT (*SIFT_consequence*) (Kumar *et al.*, 2009); PolyPhen-2 (*PPH_consequence*) (Adzhubei *et al.*, 2013); and GERP scores (*gerp_score*) (Davydov *et al.*, 2010). While well-studied viruses, such as SARS-CoV-2, potentially allow for variant conservation properties to be assessed [see variant frequencies and SIFT scores in Dunham *et al.* (2021)], we removed such features to build non-human-genome-compatible classifiers applicable to a wider variety of viruses lacking sufficient information. Furthermore, feature space reduction also facilitates downstream calculations on the limited-qubit quantum computers considered. In general, though, the methods are designed to incorporate all features given availability of sufficiently comprehensive variant databases.

In this study, we specifically investigate non-human features in the interest of applying the QNN framework to the SARS-CoV-2 3CL^*pro*^ protein. That is, we consider the subset of all features not specific to human genomes (namely everything except the GERP, PolyPhen2 and SIFT scores, and the allele frequency). This left us with nine features (*bind_site*, *distance*, *molecular_weight*, *H_bond_donor*, *H_bond_acceptor*, *rotatable_bond*, *Polar_Surface_Area*, *polarity_change_index* and *volume_change_index*), which was a tenable number for simulated QNNs. For QNNs that run on IBM’s five-qubit systems, we subselected again to those features which are not ligand-specific, leaving us with four features: *bind_site*, *distance*, *polarity_change_index* and *volume_change_index*. This is a five-qubit problem (one input qubit for each feature plus an additional readout qubit) and therefore perfectly suited to our resources. It is worth noting that, in our testing on simulated devices, we found no significant performance difference between the nine-feature group and the four-feature group. We suspect this is due to the dominance of the *bind_site* feature in the prediction process, but, regardless of the cause, it reassures us that working with the four-feature group is still a meaningful problem.

We trained various models using GenoDock’s pseudo-gold-standard training set, which was generated by applying AutoDock Vina to a large collection of co-crystal structures from the PDB, and mapping variants from germline and somatic variant databases. We then compared all methods described below [custom QNN (cQNN), qisQNN, weighted margin QNN (weighted mQNN) and GenoDock’s original random forest method] using the Platinum test set (Pires *et al.*, 2015). We applied the same models to the 6W63 mutation-ligand features. Such features were computed by GenoDock’s methodology applied to the list of SARS-CoV-2 mutations, the 6W63 crystal structure and ligand structures. Each mutation was located within the PDB structure for the target protein, and the ‘wild-type’ PDB structure of the protein was mutated at this site using the program Modeller (Webb and Sali, 2016) (resulting in a ‘mutant’ structure).

The convention used is to set the label as ‘0’ for non-disruptive mutations and ‘1’ for mutations disruptive to binding. Prior to training the cQNNs and qisQNNs, we balanced the datasets with respect to the labels, and divided the resulting dataset evenly into training/validation/testing partitions. Further information on the datasets and selection of features are provided in the Supplementary Methods.

For a more careful comparison between the classical models and quantum neural networks (QNNs), we also trained classical neural networks (NNs) with approximately matching numbers of parameters. These NNs, due to parameter-number restrictions, were built using scikit-learn’s multilayer perceptrons (scikit-learn.org/stable/modules/generated/sklearn.neural_network.MLPClassifier.html) with a single hidden layer, fully connected with the inputs. The number of nodes in this hidden layer was adjusted to approach the QNN parameter numbers, while allowing for full connections with the input layer. For example, a three-layer QNN (architecture described below) with four input features would have 4 × 3 = 12 free parameters. The classical NN would have four weight and one bias parameters per hidden layer node, and so we restricted the network to have three nodes in the single hidden layer (=15 free parameters). We note that this approach was adopted to provide a standardized method for matching the number of parameters between models, specifically suited to small network comparisons. For larger networks, specific architectural features may need to be matched beyond the parameter count, although, in general, the problem of matching quantum and classical network architectures is non-trivial.

### 2.5 Quantum neural network

Several QNN models (termed cQNN, qisQNN and weighted mQNN, varying in architectures and training) were explored to predict mutation impact on drug binding. The training set was class-imbalanced (9611 negatives and 670 positives), and each formulation had its own approach to treating such imbalance in the training set. We compared the performance of each model on a single, independent, experimentally measured dataset.

#### 2.5.1 Weighted mQNN

The weighted mQNN is based on the margin classifier recommended by PennyLane (Bergholm *et al.*, 2018; Schuld *et al.*, 2020), which is built on top of Python’s PennyLane and PyTorch frameworks. The original method is insufficient for an imbalanced dataset, and enhancement is required. In particular, we removed the random sampling of data points, and reformulated the cost function as a weighted summation of the loss function of each training point. We explored the impact of the layer and margin hyperparameters by splitting the training data in half while keeping the minority–majority ratio the same. We trained the weighted mQNN on one half of the evaluation set. Details are in the Supplementary Material.

#### 2.5.2 cQNN and qisQNN

**Fig. 2.**
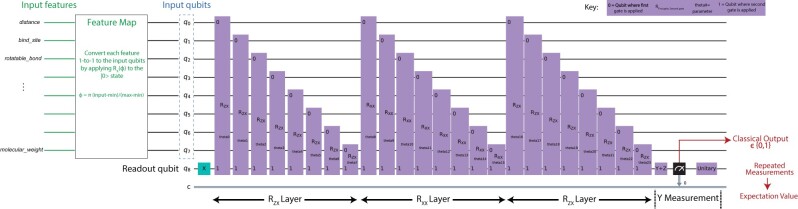
Architecture of the cQNN and qisQNN models. The number of input qubits shown here is a representative example, while in practice the number of inputs varies by the number of features considered. The initial mapping of the continuous inputs onto the interval [0,π], is not shown explicitly here

The architecture implemented herein closely follows that of Farhi and Neven (FN) (Farhi *et al.*, 2018) (example in Fig. 2). A series of features are mapped onto an equal number of input qubits, and a training dataset is used to capture structure in the data by making sure that the measurement output of the network closely matches that provided in the training data. By iteratively adjusting the free parameters in the network, we arrive at the best performing model.

As in FN, the QNN models here are variational quantum circuits, where the learned parameters are rotation phases applied to combinations of qubits and each circuit element takes the simple form of a unitary matrix rotation applied to a subset of the qubits: $U(\theta )=e^{\left(i\theta \Sigma \right)}$, where $\Sigma =\prod_{i=1}^{N_{\mathrm{qubits}}}⊗\sigma^{i}$ is a tensor product of operators from the set of 2 × 2 Pauli matrices $\left\{\sigma_{x},\sigma_{y},\sigma_{z}\right\}$ acting on a subset of the qubits. In the simplest version, the QNN circuit consists of several *input qubits* and a single *readout qubit*. The number of input qubits equals the number of features in the dataset. The qubit *z* basis is assumed throughout this discussion. Each input qubit is initialized according to the data input in the following manner: (i) we directly encode binary feature values as |0> or |1>. (ii) We apply min–max scaling, $\frac{\mathrm{input}-\min}{\max-\min}$, to continuous feature values for a range of [0,1]. Each input qubit is initialized according to the data via the application of an $R_{x}(\phi )$ operator to the |0> state, where $\phi_{i}=\pi^{*}i\mathrm{npu}t_{i}$ for the value of the corresponding data element. The readout qubit is always initialized to |1>. We considered the circuit model where, across all circuit operations, input qubits only interact with the readout qubit, and not directly with each other. This choice was made purely for investigating the effect of conditioning the output directly on each input qubit, following FN.

The interaction gates were chosen from the set of two-qubit $R_{zx}(\theta )$ and $R_{xx}(\theta )$ gates, where the first and second subscripts indicate Pauli matrices applied to a specific input qubit and the readout qubit, respectively: $R_{zx}(\theta )=e^{i\theta Z_{\mathrm{input}}⊗X_{\mathrm{readout}}}$ and $R_{xx}(\theta )=e^{i\theta X_{\mathrm{input}}⊗X_{\mathrm{readout}}}$, where $Z=\sigma_{x}$ and $X=\sigma_{x}$, and the ⊗ symbol signifies a tensor product between the operators acting on each spin’s separate subspace (the identity operator operates on all other qubits; i.e. each gate only impacts the explicitly identified qubits). A *layer* of gates is a sequence of one type of such gates applied to each of the inputs in turn.

The degree of interaction is governed by the angle *θ_n_* of the *n*th gate, and the *θ_n_* are the variable parameters through which the QNN is trained. We utilized an alternating layer structure, similar to FN: the one-layer QNN consisted of a layer of *R_zx_* gates; the two-layer QNN of *R_zx_* followed by *R_xx_* layers; the three-layer QNN of $R_{zx}-R_{xx}-R_{zx}$ layers; and the six-layer QNNs involved three alternations of *R_zx_* and *R_xx_* layers.

After all layers are executed, the *y*-component of the readout is measured, and the expectation value of that measurement is the QNN’s predicted label for the given input, $\mathrm{prediction}=<z,1|U^{†}(\vec{\theta })Y_{\mathrm{readout}}U(\vec{\theta })|z,1>$, where $U(\vec{\theta })$ is the time-ordered product of all gates $U(\theta_{n})$ in the circuit and $Y_{n+1}$ is the measurement of the *y*-component of the readout qubit. |z,1> describes input qubits initialized according to data *z* and a readout qubit initialized to |1>. Since quantum measurement collapses the state into a single value, repeated measurements (‘shots’) are required to approximate the circuit’s expectation value, given as 0×Fraction of zero measurements+1×Fraction of one measurements. In our implementation, the number of circuit executions is governed by the *shots* hyperparameter. Though a higher number of shots correspond to greater measurement accuracy, it requires more circuit executions during model training, especially during parameter update (see below). In simulation, we tested shots values ranging from 20 to 200 for the simulated results and found shots = 100 to be sufficiently accurate without dramatically increasing training time. The loss function, following FN, defined as $\mathrm{loss}(\vec{\theta },z)=1-l{\left(z\right)}^{*}p\mathrm{rediction}$, where *l*(*z*) is the true output label of the training set. This function is summed over all the data points *z* and minimized with respect to the parameters $\vec{\theta }$. In prediction tasks, the label is assigned to 0 if prediction <0.5 and 1 otherwise.

As mentioned earlier, the dataset was balanced by taking the smaller number of ‘disruptive’ data points (i.e. mutation–ligand pairs for which AutoDock Vina indicated a positive change in the binding affinity upon mutation) and randomly selecting an equal number of non-disruptive data points. This balanced dataset was then split in a 1:1:1 ratio between training/validation/testing partitions. The training of the QNNs was carried out through batch stochastic gradient descent, where batches of 20 data points were input into the circuit, and the parameter values were updated after each such batch input. A full run-through of all data points in the training set constituted a complete training epoch. The number of epochs in the training depended on the performance, after each epoch, on the validation set. In the absence of improvement of validation loss after a set number of epochs (the *patience* hyperparameter), the training procedure was stopped. We found that unlike the aforementioned original mQNN, which empirically required weighted mQNN modification for good convergence (Supplementary Fig. S7), simple batch stochastic gradient descent was sufficient here.

We have implemented two types of variational QNNs: models labeled ‘cQNN’ are built using custom wrapper functions that we defined using PyTorch in accordance with loss functions and a finite-difference-based parameter update defined by FN. Models labeled ‘qisQNN’ rely on the Qiskit’s NeuralNetworkClassifier class, which uses an L1 loss function and where the parameter update is governed by a Constrained Optimization BY Linear Approximation optimizer (qiskit.org/documentation/machine-learning/stubs/qiskit_machine_learning.algorithms.NeuralNetworkClassifier.html). The training of these models is carried out using Qiskit’s QASM simulator (qiskit.org/documentation/stubs/qiskit.circuit.QuantumCircuit.qasm.html), while a limited set of test predictions based on these trained models were run on IBM Quantum’s open access five-qubit *ibmq_bogota* system (quantum-computing.ibm.com) (feature groups were restricted to four features for these test runs). For the QASM simulator, no additional noise was added, and so the only noise source was the randomness of the individual shots. We avoided adding any noise to obtain the ideal performance of the QNNs on simulators and to enable a one-to-one comparison with the noise-free classical models. On the other hand, the prediction runs on the real quantum devices were run with 1000 shots each to ensure robust statistics amid the inherent hardware noise in these devices.

To clarify the ensuing results, we emphasize that we have run many versions of these architectures with a differing number of layers, and with differing numbers of features. The variation in the layers is done to meet the restrictions on the IBM Quantum devices, and we state so in Section 3. The variation in the number of features was done to reflect different application scenarios (human versus non-human), as described above, or to reduce the number of features down to a tractable number for the IBM Quantum devices.

Further details on the QNN training and evaluation are provided in the Supplementary Methods.

## 3 Results

### 3.1 The workflow

**Fig. 3.**
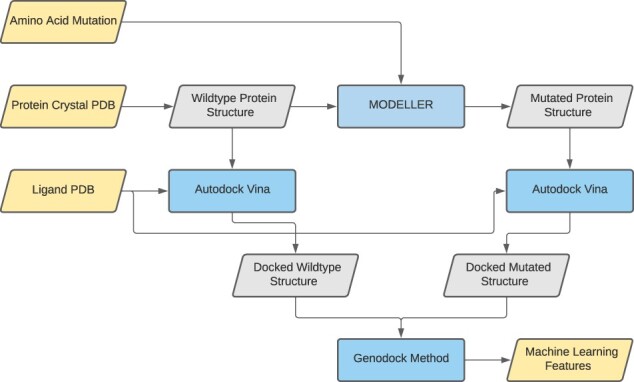
Given a combination of ligand, protein and amino acid mutation, the features used for ML are computed by comparing the docked wild-type structure and docked mutated structure computed using MODELLER and AutoDock Vina. Input and output data are represented in yellow, structure data are in gray and computational methods are in blue

We present a hybrid classical/quantum computational workflow, HypaCADD, which screens for ligands (i) that would bind favorably to a wild-type protein and (ii) that would be less likely to be affected by amino acid mutations of such a wild-type protein. Because the number of potential ligands and the number of potential mutations are both large, the number of potential ligand–protein–mutation combinations quickly becomes intractable for detailed experimental follow-up. Figure 1 illustrates the multi-step screening process, which uses docking and molecular dynamics to select a smaller number of candidates that would bind to a wild-type protein. Using the thus-selected candidates, we predict the impact of potential mutation with QML after featurization. Given a combination of ligand, unmutated protein structure and amino acid mutation, the ML featurization for predicting the impact of mutation is illustrated in Figure 3. The net result is a set of ligands that would bind to a target protein, and be robust to an input set of mutations.

### 3.2 Method validation

The paper, ‘Potential Broad Spectrum Inhibitors of the Coronavirus 3CL^*pro*^: A Virtual Screening and Structure-Based Drug Design Study’, Berry *et al.* (2015) has demonstrated an established approach to identify lead compounds, which have shown promise as inhibitors of 3CL^*pro*^ in coronavirus. To obtain confidence in our screening method, we validated our performance with 13 out of their 19 reported ligands, which had identical ZINC IDs in the version of our drug dataset. Beside the 13 ligands, 29 981 randomly selected ligands from our drug dataset were also used as background for method validation.

The same molecular docking method, AutoDock Vina, was applied to the 13 ligands from Berry *et al.* (2015) and the randomly selected ligands, by docking against the 2ZU2 receptor, a 3D structure of 3CL^*pro*^ in HCoV-229E. All the 13 reported ligands had binding affinities <−7 kcal/mol and 8 of them had binding affinities <−9.5 kcal/mol (Supplementary Table S1), which was the screening criterion used in Berry *et al.* (2015). Compared to the random ligands, the reported 13 ligands were highly enriched with ligands that can bind to the 2ZU2 receptor with high affinity (Fisher’s exact test *P*-value <2.2e-16) (Supplementary Table S2), indicating we were able to identify high-quality candidates in our initial screening for potential lead compounds.

MD simulation is a more physically realistic but computationally intensive method to assess the free energy of binding between receptors and ligands. We used the MM-GBSA calculation implemented by the GROMACS software for our MD simulations. In a 0.4 ns simulation, MM-GBSA was applied to a manageable subset of the aforementioned ligands with varying binding affinities predicted by AutoDock Vina. Specifically, based on Supplementary Table S2, 32 ligands were selected, which included all the 19 ligands having binding affinities ≤−9.5 kcal/mol and 13 ligands having binding affinities >−9.5 (with 8 selected from the random set). They were processed with MM-GBSA, as shown in Supplementary Table S3. Our comparison showed that ligands with MM-GBSA scores <−30 kcal/mol had an average of −9.5 kcal/mol AutoDock affinity score, which was significantly lower than the rest which had an average of −7.3 kcal/mol (*P*-value: 5.04E-−04). Further, we observe that the MM-GBSA binding affinity of all of Berry *et al.* (2015) ligands is significantly stronger than the aforementioned eight randomly drawn ligands with affinity >−9.5 (*P*-value: 2.7E-3, Wilcoxon rank-sum). Given these results, and the fact that the Pearson’s correlation of the AutoDock affinity score and MM-GBSA was 0.668, we suggest that the AutoDock affinity is indicative of MM-GBSA affinity. In spite of that, the accuracy of predictions is likely to further improve with more sophisticated MD simulations.

Overall, our results demonstrated that virtual screening and simulation combined can reliably serve as the foundation for predicting lead compounds for further optimization and selection.

#### 3.2.1 Defining benchmarks and accuracy of virtual screening for SARS-CoV-2 3CL^*pro*^

Recently, there have been several resolved X-ray crystallographic structures of SARS-CoV-2 3CL^*pro*^; however, most of those structures are complexed with an irreversible substrate-like inhibitor. At the time of this study, only one structure (PDB code: 6W63) represented SARS-CoV-2 3CL^*pro*^ with a reversible dipeptide inhibitor (X77). This structure and inhibitor could serve as a benchmark for virtual drug screening for SARS-CoV-2 3CL^*pro*^. To validate the docking method for SARS-CoV-2 3CL^*pro*^, AutoDock Vina and GROMACS MD were applied to the receptor and inhibitor X77 in the 6W63 structure. The root-mean square deviation between the experimental structure of X77 and best predicted pose of docking by AutoDock Vina was 0.814 Å, which was below the well-defined 2 Å benchmark to assess the accuracy of docking methodology (Wagner *et al.*, 2019). The overlay of the crystal structure of X77 and docked pose is shown in Supplementary Figure S1. The AutoDock Vina binding affinity and MM-GBSA between the receptor and inhibitor of 6W63 were −8.3 and −39.98 kcal/mol, respectively.

#### 3.2.2 Molecular docking and molecular dynamics for SARS-CoV-2 3CL^*pro*^

Confirming results of Berry *et al.* (2015) and benchmarking on the 6W63 structure provided us confidence to use AutoDock Vina and GROMACS MD as high-throughput tools for virtual screening. We further applied our protocol to SARS-CoV-2 3CL^*pro*^ against the 29 981 randomly selected ligands and 13 previously reported ligands, as aforementioned. Comparison of AutoDock results of the two receptors showed the ligands’ binding affinities to 2ZU2 and 6W63 were highly correlated but some differences existed as shown in Supplementary Figures S2 and S3, which might be due to the highly similar but not identical amino acid sequences between HCoV-229E 3CL^*pro*^ and SARS-CoV-2 3CL^*pro*^. For example, the 13 ligands highlighted in Supplementary Figure S2 shows that ligands with a high affinity (<−9.0 kcal/mol) for 2ZU2 did not have affinities as high for 6W63. There were 40 ligands with binding affinity ≤−9.5 kcal/mol for SARS-CoV-2 3CL^*pro*^, of which only four overlapped with those for HCoV-229E (Supplementary Fig. S3).

Furthermore, our MD simulations ran successfully on all the ligands having binding affinity ≤−9.5 kcal/mol and on X77 (Table 1). We surmised that a longer simulation time, such as 10 ns, would yield more stable and accurate results (though it takes a much longer runtime) and so we performed a 10 ns simulation in addition to the 0.4 ns to achieve the best results for 6W63. We observed a Spearman correlation of 0.696 (*P*-value =2.16E−7) between the 0.4 and 10 ns simulations. Results in Table 1 show that there were 13 ligands in the 10 ns simulation with a GBSA score lower than that of X77. Of these ligands, ZINC000036707984 had the lowest MM-GBSA and ZINC000016020583 had the lowest AutoDock Vina affinity score, indicating these two ligands could have a higher or comparable binding affinity to the 6W63 receptor when compared to X77. Therefore, they were selected as a manageable set of potential lead compounds for our further investigation. Along with the reference inhibitor X77, they were evaluated for the potential impacts resulting from possible variants in the 6W63 receptor.

**Table 1.** Binding affinities (≤−9.5 kcal/mol) from molecular docking using the 6W63 receptor and free energies of binding predicted by MM-GBSA that were lower than that of X77 in a 10 ns MD simulation

Ligand	VinaAffinity	GBSA 0.4ns	GBSA 10ns
ZINC000036707984	−9.7	−48.6984	−49.8774
ZINC000034758692	−9.6	−42.7249	−44.7871
ZINC000097480050	−9.6	−43.0136	−44.2139
ZINC000096114211	−9.6	−43.2604	−44.1934
ZINC000096115318	−9.9	−36.6151	−43.1172
ZINC000035424775	−9.6	−37.8856	−42.7367
ZINC000230129735	−10.3	−43.2664	−42.4759
ZINC000016020583	−10.4	−38.5203	−41.8849
ZINC000018266226	−10.4	−38.262	−41.6461
ZINC000096115318	−9.9	−44.3969	−41.6005
ZINC000035851646	−9.5	−31.9911	−41.5783
ZINC000032205245	−9.7	−38.9963	−40.9523
ZINC000016001299	−10.1	−36.6569	−40.5696
X77	−8.3	−41.5873	−39.9814

#### 3.2.3 Evaluation of virus mutations’ impact on the interactions of the lead compounds with SARS-CoV-2 3CL^*pro*^

GenoDock (Wang *et al.*, 2019) is a hybrid physical-statistical classifier to predict the impacts of variants on protein–drug interactions using genomic, structural and chemical features; however, due to the focus on human proteins, some of the features used in the published GenoDock classifier were human-specific and not applicable to viral genomes. In order to make the GenoDock framework applicable to non-human genomes, such as viruses and to further improve the predictive performance, we made several modifications and improvements including removing human-specific features (all the conservation scores and allele frequencies), feature normalization and making use of an ensemble method. The final set of features employed are: volume change, polarity change, distance, molecular weight, H-bond donor, H-bond acceptor, rotatable bond, polar surface area and bind site True/False. Table 2 demonstrates that the new virus-genome-compatible classifier with random forest implementation had comparable performance compared to the original GenoDock classifier with random forest implementation based on a 10-fold cross-validation.

**Table 2.** Comparison of the original GenoDock classifier and new classifier, which is virus-genome compatible after removing human-specific features

	Original	Human-specific features removed
Accuracy	0.965 (0.005)	0.964 (0.006)
*F*1	0.701 (0.040)	0.705 (0.047)
Recall	0.640 (0.050)	0.663 (0.061)
Precision	0.779 (0.054)	0.756 (0.056)

*Note*: Both classifiers were implemented using the random forest machine-learning model. The numbers in parentheses are standard deviations of the performance scores.

#### 3.2.4 Classical computing model performance on external validation set

We utilized Genodock’s 10,281 ‘pseudo-gold-standard’ training points based on the use of AutoDock Vina to quantify the impacts of amino acid variations on ligand binding, as well as 86 validation data points from the independent Platinum database (Pires *et al.*, 2015) for our non-human-feature classifiers. The Platinum database results originate from experimental measurements of mutation effects. We retrained a model using GenoDock’s class-balanced random forest method with the pseudo-gold-standard set, and evaluated against the Platinum validation set. The classification method yielded an AUC of 0.628, which was on par with the original publication. Given the relative scarcity of experimental data measuring the impacts of amino acid mutation on ligand binding, and our consequent training on a computational binding affinity dataset, we see this result as providing reasonable validation for our method. We, however, hope that the future use of experimental data to train models would further improve the agreement.

#### 3.2.5 QC model performance on external validation set

Multiple formulations of QNNs have been explored to compare the performance and practicality of customized versus published formulations. While the primary focus of this article is on demonstrating the application of our computational workflow on SARS-CoV-2 proteins, we also trained our cQNN and qisQNN models on all the feature groups of the original GenoDock publication, including human-specific features, such as conservation scores. Our results on the GenoDock and Platinum datasets (Supplementary Figs S4 and S5, respectively) indicate very similar performance of three-layer QNNs with the random forest model (in terms of AUC values). In all cases, the performance of the (approximately parameter-number-matched) classical NNs was on par with the best QNN model. With this confidence in the performance of the QNNs on a more general set of feature groups, we proceeded to apply the cQNN and qisQNN models to the non-human feature group relevant to the SARS-CoV-2 protein. These models performed very well on a GenoDock test set (Supplementary Fig. S6). The results for the Platinum dataset below are from three-layer cQNNs and qisQNNs.

The weighted mQNN (see Section 2) is a reformulation of PennyLane’s margin classifier (unweighted and trained on 500 random samples from training set per iteration). Non-human features from Genodock are fit into a four-qubit formulation. Due to the high computational cost, we trained the model with one half of the pseudo-gold-standard training set, then validated on the hold-out part of the training set. Supplementary Figure S7 shows the convergence of the training process. It reveals that the weighted cost function stabilized the convergence when compared to the unweighted. The classification performance is demonstrated in Supplementary Figure S8. It shows that the mQNN could achieve an AUC as high as 0.937 on the validation set.

**Fig. 4.**
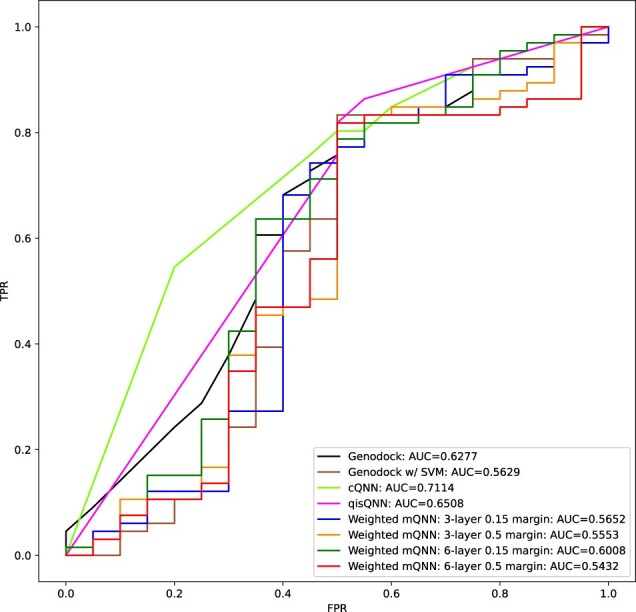
Receiver operating characteristic evaluated on the 86 validation data points from the Platinum database

We applied our quantum modeling methods with quantum simulators to the Platinum dataset for the comparisons in Figure 4 and Table 3. Figure 4 shows the true positive rate versus false positive rate, where positive calls were made by applying different thresholds to measure, e.g. the *z*-projection of output qubit. We note that the density of points for each curve can vary depending on the number of tie scores (Supplementary Table S4). Table 3 shows the *F*1, sensitivity and precision of binary prediction by the model, as well as the AUC (Supplementary Methods S3a), which is a binary-threshold-invariant measurement of model performance. The AUC indicates the sensitivity–specificity trade-offs if one wants to, e.g. increase specificity at the cost of sensitivity. We also note that the precision was calculated from binary calls under heavy class imbalance in favor of positive labels, as discussed in the Supplementary Material.

**Table 3.** Sensitivity, precision, specificity and *F*1 score of the models with default classification thresholds

Method	AUC	*F*1	Sensitivity	Precision
GenoDock	0.63	0.83	0.88	0.79
GenoDock w/SVM	0.56	0.83	0.82	0.84
cQNN	0.71	0.82	0.80	0.84
qisQNN	0.65	0.82	0.80	0.84
Weighted mQNN: 3 l, 0.15 m	0.57	0.80	0.77	0.84
Weighted mQNN: 3 l, 0.5 m	0.56	0.83	0.82	0.84
Weighted mQNN: 6 l, 0.15 m	0.60	0.82	0.80	0.83
Weighted mQNN: 6 l, 0.5 m	0.54	0.83	0.82	0.84

*Note*: l and m denote layer and margin setting.

The cQNN method had higher AUC and sensitivity in the high specificity regime compared to other methods, classical or quantum that we considered here. Importantly, these models designed for near-term quantum devices performed on par with GenoDock, a classical model that was not reduced in its complexity for these comparisons. As an additional corroboration of the quality of the results, we also trained a classical dense NN, which was approximately matched in terms of the number of parameters. The classical NN (Supplementary Fig. S9) performed well, showing an AUC of 0.68. This demonstrates that specialized QNNs have, at least, a representational power that is comparable to classical models for the current dataset. However, we did notice that the performance of the QNN models on the external validation set was heavily correlated with a single variable: *bind_site*, a binary indicator variable labeling whether a mutation occurs within the binding site of a ligand or not. For example, if the mutation was experimentally observed to be disruptive but did not occur in the binding site, the cQNN and qisQNN models would incorrectly label the data point as non-disruptive. This was also borne out in the cross-feature group comparisons we conducted using the original GenoDock and Platinum datasets (Supplementary Figs S4 and S5), where feature groups that included the *bind_site* and *distance* parameters (both of which are related) performed better. The outsize influence of these variables on the performance of the models was also found in the GenoDock analysis (Wang *et al.*, 2019), but does not imply the absence of influence by other features as seen in the original publication. There are some subtle differences in performance between the different feature groups that all include *bind_site* as a feature. It is likely, however, that fully capturing more complex cross-feature interactions would need more qubits and more complex gate architectures. For the mQNN, we see definite performance improvement when class imbalance is addressed by the weighted cost function. We see increased performance when the network is increased from 3 to 6 layers and margin is decreased from 0.5 to 0.15, and when class imbalances are compensated by weighting of the loss function.

**The question of QC advantages:** We do not claim the positive results observed for the cQNN and qisQNN as definitive evidence of QC advantages. There are slight differences in the performance of GenoDock and the QNNs on the Platinum dataset predictions: as mentioned above, the QNNs seem to heavily depend on the *bind_site* variable, sometimes leading to incorrect predictions; GenoDock’s predictions also carry a strong dependence on *bind_site*, but deviate in a small number of cases from being completely determined by this variable. This means that the trained representations of the underlying probability distributions of outputs conditional on inputs are different, without any clear supremacy of one model over the other. We see our approach here as *priming* the problem for more complex QC solutions. That is, provided with this particular way of contending with the problem of mutational impact quantification, we foresee extensions to the model that may lead to more parsimonious representations of the data structure, through improvements in computational complexity (smaller numbers of operations than classical counterparts) or model complexity (a richer model in QC for the same numbers of parameters). Examples of ideas being considered include the use of quantum embedding kernels (Lloyd, 2020) to allow non-linearities in the representation of data, and amplitude encoding (e.g. Schuld *et al.*, 2018) to possibly compress a higher dimensional problem into a smaller number of qubits. With the formulation provided here as a foundation, we hope that heuristic evidence of QC advantages and interesting differences in representation might be forthcoming in further work in this area.

#### 3.2.6 SARS-CoV-2 variant impact predictions in simulation and on real QC devices

With the quantum formulation established to be at least on par with non-quantum methods, we applied the models to predict the impact of amino acid substitution in the SARS-CoV-2 virus 3CL^*pro*^ on protein–ligand binding. As of January 2021, 98 amino acid substitution variations had been detected in SARS-CoV-2 3CL^*pro*^ (Supplementary Fig. S10). The ligand candidates were chosen to be the X77 ligand for its known crystalized structure with 6W63, ZINC000016020583 for its lowest docking affinity and ZINC000036707984 for its lowest MM-GBSA value (Table 1).

Because of the scarcity of quantum computer time, we trained all models using quantum simulators. We subsequently established consistency between test set prediction results when executed on quantum simulators and on real quantum computers. Again, addressing resource limitations, we separately trained one- and two-layer cQNNs on simulators and used those for prediction on the real devices. We selected, by Vina docking affinity change estimation, three mutations that would likely cover some positive and negative calls for the ligands, and compared the predictions from simulators and real quantum computers. Table 4 shows that cQNN, the best performing model, was consistent between simulation and 1000-shot measurement on an IBM five-qubit device (the *ibmq_bogota* system) with a quantum volume of 32. Interestingly, we had to increase the number of shots relative to the simulation results in order to get robust statistics. Additionally, we found that the noise in the output expectation values increases with an increase in the number of layers, implying that each set of new gates added to a circuit brings further noise. This can be seen in Supplementary Table S5, where in addition to the same predicted output values as in Table 4, we included the expectation values output by one- and two-layer circuits (and thresholded at 0.5 to determine the final output label of 0 or 1). With two layers, the expectation values appear to cluster closer to the threshold of 0.5, relative to the one-layer case. In summary, while the expectation is that simulated values will deviate from real device-generated results due to decoherence and noise in the real devices, for the small number of data points considered, we find no difference in predictions. However, we see the effects of device noise in the expectation values observed and in the number of shots required for robust statistics.

**Table 4.** Comparison of outputs between simulation and real quantum computer on the 3CL^*pro*^ mutations

				cQNN	cQNN	cQNN	Weighted mQNN	Weighted mQNN	Weighted mQNN
					One-layer	Two-layer	Six-layer, 0.15 margin	Six-layer, 0.15 margin	Six-layer, 0.15 margin
					Five-qubit IBM	Five-qubit IBM	PennyLane	DM1	Rigetti
Position	Ref	Alt	Ligand	Simulation	1000 shots	1000 shots	Simulation	1000 shots	1000 shots
160	CYS	PHE	X77	0	0	0	1	1	0
168	PRO	SER	X77	1	1	1	1	1	0
188	ARG	SER	X77	1	1	1	1	1	0
160	CYS	PHE	ZINC000016020583	0	0	0	1	1	1
168	PRO	SER	ZINC000016020583	1	1	1	1	1	0
188	ARG	SER	ZINC000016020583	1	1	1	1	1	0
160	CYS	PHE	ZINC000036707984	0	0	0	0	0	0
168	PRO	SER	ZINC000036707984	1	1	1	1	1	0
188	ARG	SER	ZINC000036707984	1	1	1	1	1	0

*Note*: Predictions are indicated as 0 or 1 depending on whether a mutation is predicted to be non-disruptive or disruptive to binding of the ligand, respectively. The results shown include: a three-layer (FN architecture) cQNN circuit where both training and predictions were run using the QASM simulator; and one- and two-layer cQNN circuits trained on the QASM simulator, but with prediction runs on an IBM Quantum five-qubit system (marked by the term ‘real’), with 1000 shots in each case; a weighted mQNN circuit with predictions conducted through simulation (default Pennylane simulator with six-layer and 0.15 margin); a six-layer, 0.15 margin, weighted mQNN circuit with predictions conducted through runs on an AWS’s Braket DM1 system, 1000 shots; a six-layer, 0.15 margin, weighted mQNN circuit with predictions conducted through runs on a Rigetti system, 1000 shots.

We also observed that, unlike the IBM platform, the simulated values produced by PennyLane’s analytical simulator agreed poorly with those produced on the Rigetti Aspen-10 hardware. To understand this, we used the AWS’s SV1 and DM1 simulators to perform 1000-shot simulation with the PennyLane framework, and the SV1 and DM1 results agreed with those of PennyLane’s simulator. We also used the AWS’s Braket API to perform simple individual qubit RX rotations followed by *z*-direction measurements as well as to perform pairwise CNOT operations followed by *z*-direction measurements. Using the *disable_qubit_rewiring* option in the API, we disabled automatic reassignment of the API’s logical qubits onto physical qubits, and we observed that the *z*-direction measurements deviated from simulation/analytical values on a physical qubit-by-qubit basis, and the amount of deviation could be as much as one order of magnitude from one physical qubit to another. This qubit-dependent, heterogeneous noise could in principle be mitigated by a weighted mQNN with four qubits and limited nearest-neighbor CNOT operations, which maps well onto the nearest-neighbor physical connectivity of the Rigetti system (e.g. qubit 1/2/15/16 in Supplementary Fig. S11). However, the encoding of 10 features into four qubits is performed by an extra sequence of gate operations (Mottonen *et al.*, 2005) including additional pairwise connectivity that is not present in the Rigetti’s topology if the operations were to be restricted to involve only four qubits. From AWS’s reported metadata, we observed that the PennyLane four-qubit circuit was transpiled into operations with a higher number of gates and operations before execution on the Rigetti machine. We further confirmed this by modifying the *aws-pennylane* plugin to disable qubit reassignment and transpilation, which induced an infrastructure error about missing two-qubit coupling for the CNOT operations (confirmed to be the ones missing from the hardware) that were required to encode 10 features into four qubits. While the PennyLane framework can indeed produce consistent results among all three simulators, actual execution of non-trivial circuits on real physical hardware, such as Rigetti might be hampered by qubit-dependent, heterogeneous noise. We observed that cQNN, developed within the IBM ecosystem, has the best reproducibility between simulated and real quantum simulation.

Having studied the correlations between real and simulated quantum computation, we make more predictions with the quantum simulator. The models’ predictions are listed in Supplementary Table S6. For binary prediction over all mutations and ligands, the cQNN and qisQNN had 100% concordance and the cQNN and Genodock had 99.7% concordance, whereas cQNN and weighted mQNN had 75.2% concordance. To note, X77 was predicted to be susceptible to the mutations VAL20ILE and ILE43VAL, while ZINC000016020583 was predicted to be not affected; furthermore, ZINC000016020583 had stronger binding tendency according to both docking and 10 ns molecular dynamics simulation (Table 1).

## 4 Discussion

We establish a hybrid system of classical and QC to advance CADD. It utilizes well-established virtual screening methods to identify lead compounds. It leverages ML to build an improved physical-statistical classifier capable of predicting the effect of variants from hypothetical and real mutations in receptor sequences of any genome to provide insights into drug efficacy. From screening to lead identification and to variant effect prediction, our approach can work not only in classical computing, but also in conjunction with QC to eventually go beyond the current limits of classical computers. We demonstrated its performance and capability by applying the system to viral genomes with QML.

Antiviral drugs targeting SARS-CoV-2 3CL^*pro*^ could help to fight against the COVID-19 pandemic. As a proof of concept, we validated our screening method with the HCoV-229E 3CL^*pro*^ protein. We then applied our system to the SARS-CoV-2 3CL^*pro*^ protein, where two lead compounds, ZINC000016020583 and ZINC000036707984, were identified as potential inhibitors from 30 000 compounds subsampled from a drug dataset of over 11 million compounds. The new coronavirus is known to mutate frequently, sometimes with serious consequences in terms of immune evasion. It is essential to efficiently and reliably predict the effect of the mutations on any target drugs in the design process and to know the corresponding efficacy of a candidate drug. We therefore further applied our improved method of variant effect prediction to the identified compounds by using ML and both classical and QC techniques, demonstrating that the performance of QC is on par with classical computing. Specifically, QML models have been shown to have sufficient representational power to capture the structure in large-scale biological data. Our results showed indicative and insightful results based on the known mutations thus far, proving the utility of our approach for an advanced, promising and robust drug design.

With the rapid advancement of computer power and algorithms, ML has become an indispensable tool to discover patterns in large-scale, high-dimensional data. We explored the emerging field of QML, which is the interplay of quantum computers and ML. Because of its exploitation of operations in the $2^{N}$-dimensional Hilbert space (for *N* qubits), QC can solve certain problems that are hard for classical computers [quantumalgorithmzoo.org (Shor, 1997; Van Dam *et al.*, 2006)]. In this article, we demonstrated several QML formulations trained on data that was highly imbalanced and was of computational origin, and then benchmarked their performance on test data of experimental origin. The limited, orthogonal Platinum test data showed that the QML models performed at least on par with the established classical ML methods trained on the same dataset. Lastly, we assessed the fidelity of quantum simulators with respect to real quantum computers. Our classical-quantum hybrid approach demonstrated how QC can be combined with classical computing robustly, with significant potential for added value in complex tasks, such as protein-ligand binding predictions. As quantum computers become more powerful and stable, and more quantum-specific algorithms become available, we foresee that the utility of our approach will grow. For example, QC can be used for protein design/folding (Perdomo *et al.*, 2008; Perdomo-Ortiz *et al.*, 2012), improving QM/MM calculation in molecular docking (Arodola *et al.*, 2017), modeling with neural-network quantum states (Choo *et al.*, 2020), as well as many other essential computational tasks.

## Supplementary Material

btac789_Supplementary_Data

## Data Availability

The data underlying the QNN analysis are available with the Jupyter Notebooks for academic use on GitHub: https://www.github.com/hypahub/hypacadd_notebook. The datasets were derived from sources in the public domain: drug dataset from zinc.docking.org, receptor structures from rcsb.org, SARS-COV-2 mutations from covidcg.org, Genodock dataset from the supplmenental material of Wang *et al.*, 2019.
